# Effects of clinically relevant acute hypercapnic and metabolic acidosis on the cardiovascular system: an experimental porcine study

**DOI:** 10.1186/cc13173

**Published:** 2013-12-30

**Authors:** Milan Stengl, Lenka Ledvinova, Jiri Chvojka, Jan Benes, Dagmar Jarkovska, Jaromir Holas, Patrik Soukup, Jitka Sviglerová, Martin Matejovic

**Affiliations:** 1 Department of Physiology, Faculty of Medicine in Pilsen, Charles University in Prague, Pilsen, Czech Republic; 2 First Medical Department, Faculty of Medicine in Pilsen, Charles University in Prague, Pilsen, Czech Republic; 3 Department of Anesthesiology and Critical Care Medicine, Faculty of Medicine in Pilsen, Charles University in Prague, Pilsen, Czech Republic; 4 Biomedical Center, Faculty of Medicine in Pilsen, Charles University in Prague, Pilsen, Czech Republic

## Abstract

**Introduction:**

Hypercapnic acidosis (HCA) that accompanies lung-protective ventilation may be considered permissive (a tolerable side effect), or it may be therapeutic by itself. Cardiovascular effects may contribute to, or limit, the potential therapeutic impact of HCA; therefore, a complex physiological study was performed in healthy pigs to evaluate the systemic and organ-specific circulatory effects of HCA, and to compare them with those of metabolic (eucapnic) acidosis (MAC).

**Methods:**

In anesthetized, mechanically ventilated and instrumented pigs, HCA was induced by increasing the inspired fraction of CO_2_ (*n* = 8) and MAC (*n* = 8) by the infusion of HCl, to reach an arterial plasma pH of 7.1. In the control group (*n* = 8), the normal plasma pH was maintained throughout the experiment. Hemodynamic parameters, including regional organ hemodynamics, blood gases, and electrocardiograms, were measured *in vivo*. Subsequently, isometric contractions and membrane potentials were recorded *in vitro* in the right ventricular trabeculae.

**Results:**

HCA affected both the pulmonary (increase in mean pulmonary arterial pressure (MPAP) and pulmonary vascular resistance (PVR)) and systemic (increase in mean arterial pressure (MAP), decrease in systemic vascular resistance (SVR)) circulations. Although the renal perfusion remained unaffected by any type of acidosis, HCA increased carotid, portal, and, hence, total liver blood flow. MAC influenced the pulmonary circulation only (increase in MPAP and PVR). Both MAC and HCA reduced the stroke volume, which was compensated for by an increase in heart rate to maintain (MAC), or even increase (HCA), the cardiac output. The right ventricular stroke work per minute was increased by both MAC and HCA; however, the left ventricular stroke work was increased by HCA only. *In vitro*, the trabeculae from the control pigs and pigs with acidosis showed similar contraction force and action-potential duration (APD). Perfusion with an acidic solution decreased the contraction force, whereas APD was not influenced.

**Conclusions:**

MAC preferentially affects the pulmonary circulation, whereas HCA affects the pulmonary, systemic, and regional circulations. The cardiac contractile function was reduced, but the cardiac output was maintained (MAC), or even increased (HCA). The increased ventricular stroke work per minute revealed an increased work demand placed by acidosis on the heart.

## Introduction

Acidosis is a dominant type of acid–base disturbance in critical illness that has been reported to influence a vast number of physiological and pathophysiological processes [1,2], including the complex regulation of cardiovascular functions. In the systemic circulation, respiratory acidosis is known to reduce left ventricle contractility [3]. However, this is compensated for by an increased heart rate and a reduced systemic vascular resistance, resulting in an increased cardiac output. The direct effects of acidosis in the heart have been studied in great detail, because local acidosis usually accompanies ischemia. A number of detrimental effects of myocardial ischemia, such as the negative inotropic effect or induction of arrhythmias, may be attributed to acidosis [4,5]. The negative inotropic effect of acidosis has long been known [6], and a number of mechanisms involved in this phenomenon have been put forward. Although acidosis has been reported to affect virtually every step in excitation-contraction coupling, it is now generally accepted that the negative inotropic effect of acidosis is predominantly related to a decreased sensitivity of contractile proteins to Ca^2+^[7-9]. It should be emphasized that, in general, studies on the effects of acidosis in the heart have been performed mostly in isolated tissues or myocytes, and the pH values used were rather low (~6.0 to 6.5), corresponding to local acidosis in cardiac tissue during ischemia (for example, see [10]).

The effects of acidosis on pulmonary circulation are less clear. For a long time, a general consensus suggested that hypercapnic acidosis (HCA) increases the pulmonary vascular resistance, the effect being attributed to acidosis that overrules the direct vasodilator effect of CO_2_ (for example, [11,12]). This traditional view was supported by a more recent study in healthy humans showing a vasoconstrictor pulmonary vascular response to hypercapnia [13]. In contrast, in the isolated rabbit lung, no effect of HCA on normoxic pulmonary vascular tone was found, although it was able to increase the constrictor response to hypoxia [14]. Finally, acute acidosis may also affect a number of extrapulmonary organ systems; however, direct data on the effects of acidosis on systemic blood-flow distribution and extrapulmonary organ-related circulations are very limited.

The HCA may also have therapeutic implications. In acute respiratory distress syndrome (ARDS), the ventilation strategies that reduce lung stretch have been demonstrated to decrease morbidity and mortality in patients with ARDS [15-18]. This protective ventilation is usually associated with hypercapnia, which was, in a classic view, regarded as a tolerable side effect (permissive hypercapnia) [19]. A growing number of studies on the effects of HCA, both in experimental models and in patients, indicate that instead of being a tolerable adverse effect, HCA may exert direct protective actions and may be therapeutic by itself [1].

In general, a lack of integrative studies have been performed in clinically relevant large-animal models that would address the cardiovascular effects *in vivo*, simultaneously, in systemic, regional, and pulmonary circulations, to determine which of the cellular and organ effects may dominate and have clinical consequences. The majority of experimental studies do not include the investigation of the effects of acidosis independent of other confounding factors. Therefore, to separate the effects of acidosis from the effects of an underlying disease, as seen in a clinical setting, we performed a highly complex physiological study in healthy pigs, to evaluate the systemic and organ-specific biologic effects of clinically relevant metabolic and hypercapnic acidosis in several vital organs. Because it is not clear whether the effects of HCA are due to the hypercapnia or to the acidosis *per se*, and an increasing body of evidence suggests that acidosis may be the critical factor [20], the effects of HCA were compared with those of metabolic acidosis (MAC).

Finally, the effects of acidosis were tested in isolated cardiac tissues to discriminate the direct primary cardiac effects from those induced secondarily by the vascular actions of HCA.

## Materials and methods

Animal handling was in accordance with the European Directive for the Protection of Vertebrate Animals Used for Experimental and Other Scientific Purposes (86/609/EU). The experiments were approved by the Committee for Experiments on Animals of the Faculty of Medicine in Pilsen, Charles University, in Prague. Twenty-four domestic pigs, of both sexes and of similar weights (37 ± 1 kg), were assigned in a fixed order (1:1:1) into three groups: control group (*n* = 8), MAC group (*n* = 8), and HCA (*n* = 8) group.

### Anesthesia and instrumentation

The experimental animals were kept fasting for 18 hours before the experiment, with unlimited access to water. Anesthesia was induced with ketamine (2 mg/kg IM), azaperone (2 mg/kg IM), and propofol 2% (1 to 2 mg/kg IV). The animals were mechanically ventilated (FiO_2_, 0.4; PEEP, 5 cm H_2_O; tidal volume, 10 ml/kg), and the respiratory rate was adjusted to maintain end-tidal pCO_2_ between 4.0 and 5.0 kPa, except in the HCA group, in which the respiratory rate was unchanged over the time of the experiment (FiO_2_ was adjusted to maintain SaO_2_ above 90%). During the instrumentation, surgical anesthesia was maintained with continuous intravenous thiopental (10 mg/kg/h) and fentanyl (10 to 15 μg/kg/h), and then deescalated to a continuous thiopental (5 mg/kg/h) and fentanyl infusion (5 μg/kg/h), which was maintained until the end of the experiment. Muscle paralysis was achieved with pancuronium (0.2 mg/kg/h). An infusion of Plasma-Lyte solution (Baxter Healthcare, Deerfield, IL, USA) at 15 ml/kg/h was administered during the surgical procedures, and then reduced to 7 ml/kg/h as a maintenance fluid.

A central venous catheter (Certofix Trio V715; B Braun, Melsungen, Germany) for drug and fluid infusion was inserted through the left jugular vein. A balloon-tipped thermodilution pulmonary artery catheter (Corodyn Thermodilution Infusion Catheter; B Braun) was placed via the right jugular vein. A femoral arterial catheter was placed for blood pressure monitoring and blood sampling, and a fiberoptic catheter (COLD Z-021; Pulsion Medical, München, Germany) was placed for thermal-dye double-indicator dilution measurements. A midline laparotomy was performed, and precalibrated ultrasound transit-time flow probes (Transonic Systems, Ithaca, NY, USA) were placed on the portal vein, common hepatic artery, left renal artery, and left carotid artery. Venous catheters (Certofix Duo V715; B Braun) were introduced into the portal, renal, hepatic, and jugular veins for the determination of pH, pO_2_, pCO_2_, and for hemoglobin oxygen saturation.

### Hemodynamic measurements

The cardiac output (CO) was determined by thermodilution (66S Monitor; Hewlett Packard, Palo Alto, CA, USA), and the data reported as the mean of three injections of 10 ml of ice-cold saline, randomly spread over the respiratory cycle. The intrathoracic and end-diastolic blood volume were measured by arterial thermal-green dye double-indicator dilution (COLD Z-021; Pulsion) after the injection of 10 ml (2.5 mg/ml) of cold indocyanine green (Pulsion Medical).

### Experimental protocol

HCA was induced by increasing the inspired fraction of CO_2_ in two steps. After the 1-hour baseline period (arterial pCO_2_, 4.5 to 5.0 kPa, collection of baseline data), the inspired fraction of CO_2_ was increased to achieve hypercapnic acidosis with an arterial pH of 7.25 (arterial pCO_2_ , about 10 kPa). After 60 minutes of a steady state, the inspired fraction of CO_2_ was further increased to achieve a pH of 7.1 (arterial pCO_2_, about 15 kPa), which was again maintained for 60 minutes before the collection of data (hemodynamic data, electrocardiogram). The arterial blood-gas analysis was performed every 15 minutes to determine the degree of acidosis.

MAC was induced by administering a continuous IV infusion of HCl (2 *M*) at a rate of 10 ml/h. The same two-step experimental schedule as for respiratory acidosis was used: the first step to an arterial pH of 7.25 was followed by the second step to an arterial pH of 7.1. The arterial pCO_2_ was maintained constant. To achieve the target arterial pH, the infusion rates of HCl were adjusted individually.

The experimental protocols in the control animals were similar to those in the groups for respiratory and metabolic acidosis, except for the plasma pH manipulation. The electrocardiogram (lead II) was recorded by using the BIOPAC system (BIOPAC Systems, Inc., Santa Barbara, CA, USA). The QT_c_ (QT corrected for heart rate) values were calculated by using the Fridericia formula (QT_c_ = QT/RR^1/3^). For the heart-rate variability (HRV) analysis, the RR intervals were detected, and the HRV parameters (statistical parameters, frequency domain parameters, and Poincaré plots parameters) were computed automatically from 5-minute intervals by using custom measurement and analysis Matlab routines.

### *In vitro* experiments

At the end of the *in vivo* experiment, the heart was quickly excised and transported in cold Ca^2+^-free Tyrode solution to the electrophysiological laboratory. Trabeculae (diameter about 1 mm) were dissected from the right ventricle, placed into an experimental chamber, and attached to an isometric-force transducer (F30; Hugo Sachs, March-Hugstetten, Germany). The preparation was perfused with warm (36°C) oxygenated Tyrode solution at a constant flow rate (6 to 10 ml/min). After a stabilization period (>30 minutes), the steady-state contractions and action potentials (stimulation frequencies of 0.5, 1, and 2 Hz) were recorded. The time course of the contraction-relaxation cycle was characterized by using the time-to-peak (time from resting tension to the peak of contraction) (TTP) and the time-to-90%-relaxation (R_90_). The resting tension was taken as zero, and the membrane potential was measured with glass microelectrodes (filled with 3 *M* KCl; resistance, >20 MΩ). The APD was measured at the 50% and 90% levels of repolarization (APD_50_, APD_90_). All intervals, durations, and amplitudes were measured in five beats, averaged, and the mean values were used for further analyses and comparisons. Data were recorded and analyzed by using the National Instruments data-acquisition hardware and software (National Instruments, Austin, TX, USA).

### Solutions and chemicals

The composition of the Tyrode solution was (in m*M*): NaCl, 137; KCl, 4.5; MgCl_2_, 1; CaCl_2_, 2; glucose, 10; HEPES, 5; and pH adjusted to 7.4 with NaOH (or to 7.1). All chemicals were purchased from Sigma Aldrich.

### Statistical analysis

Data are presented as the mean ± SD. After testing for the normality of the distribution, statistical comparisons were made with the paired or unpaired Student *t* test, one-way ANOVA, and two-way repeated-measures ANOVA followed by the Bonferroni test, by using the OriginPro 8.5 software (OriginLab Corporation, Northampton, MA, USA). Differences at p ≤ 0.05 were considered to be significant.

## Results

In the control group, the experimental procedure did not influence the blood pH values (Table 1). Both the HCA and MAC interventions successfully decreased the arterial blood pH values to 7.1, as planned (Table 1). In HCA, the partial pressures of CO_2_ in both the arterial and venous blood were increased (Table 1). In MAC, a moderate increase in the partial pressure of CO_2_ in the venous blood developed (Table 1).

**Table 1:** Blood parameters

	**Control**		** Metabolic acidosis**			**Hypercapnic acidosis**			
	**Baseline**_**st**_	**Baseline**_**end**_	**Δ**	**Baseline**	**Acidosis**	**Δ**	**Baseline**	**Acidosis**	**Δ**
P_a_CO_2_	5.39 ± 0.85	5.17 ± 0.68		5.34 ± 0.79	5.8 ± 1.08		5.04 ± 0.5	15.57 ± 1.37	↑
P_v_CO_2_	7.09 ± 1.4	6.46 ± 0.83		6.73 ± 0.66	7.97 ± 1.15	**↑**	6.14 ± 0.38	17.19 ± 1.89	↑
P_a_O_2_	16.7 ± 3.72	14.85 ± 2.85		16.71 ± 3.7	16.61 ± 4.11		17.74 ± 6.38	15.17 ± 1.2	
P_v_O_2_	4.01 ± 0.38	3.94 ± 0.52		3.96 ± 0.43	6.03 ± 0.8	↑	4.21 ± 0.88	7.74 ± 0.88	↑
pH_a_	7.52 ± 0.06	7.53 ± 0.05		7.52 ± 0.03	7.10 ± 0.01	↓	7.56 ± 0.01	7.10 ± 0.02	↓
pH_v_	7.45 ± 0.06	7.47 ± 0.04		7.46 ± 0.02	7.03 ± 0.02	↓	7.50 ± 0.02	7.06 ± 0.02	↓
$\mathrm{HC}O_{3}{}_{a}^{–}$	31.68 ± 2.53	29.84 ± 7.29		34.14 ± 4.18	13.1 ± 2.17	↓	33.5 ± 3.21	34.7 ± 2.32	
$\mathrm{HC}O_{3}{}_{v}^{–}$	35.88 ± 2.45	31.52 ± 7.52		35.11 ± 2.9	15.19 ± 1.64	↓	34.84 ± 2.26	35.8 ± 2.47	
Hb	86.63 ± 10.2	85.63 ± 13.55		80.5 ± 12.97	89.5 ± 13.93	**↑**	81.75 ± 8.38	103.5 ± 9.87	**↑**
Sat_a_	99.26 ± 1.46	98.7 ± 2		99.18 ± 1.27	95.8 ± 3.81	↓	98.94 ± 1.32	94.38 ± 2.8	↓
Sat_v_	49.26 ± 7.48	47.13 ± 4.06		49.11 ± 8.44	49 ± 6.25		50.13 ± 7.76	63.75 ± 2.18	**↑**
BE_a_	9.53 ± 2.67	9.83 ± 2.04		10.48 ± 4.07	−16.56 ± 2.26	↓	11.73 ± 3.06	5.71 ± 2.19	↓
BE_v_	12.3 ± 2.39	10.29 ± 3.76		11.89 ± 3.12	−15.5 ± 1.76	↓	12.11 ± 2.22	6.08 ± 2.26	↓

↑, significantly increased, *P* < 0.05; ↓, significantly decreased, *P* < 0.05; P_a_CO_2_, arterial partial pressure of CO_2_ (kPa); P_v_CO_2_, venous partial pressure of CO_2_ (kPa); P_a_O_2_, arterial partial pressure of O_2_ (kPa); P_v_O_2_, venous partial pressure of O_2_ (kPa); pH_a_, arterial pH; pH_v_, venous pH; $\mathrm{HC}O_{3}{}_{a}^{-}$, $\mathrm{HC}O_{}{}_{3}^{–}$ concentration in arterial blood (mM); $\mathrm{HC}O_{3}{}_{v}^{-}$, $\mathrm{HC}O_{}{}_{3}^{–}$ concentration in venous blood (mM); Hb, hemoglobin concentration (g/L); Sat_a_, hemoglobin saturation in arterial blood (%); Sat_v_, hemoglobin saturation in venous blood (%); BE_a_, base excess in arterial blood (mM); BE_v_, base excess in venous blood (mM). The venous blood refers to mixed venous blood sampled from the pulmonary artery.

**Figure 1:**
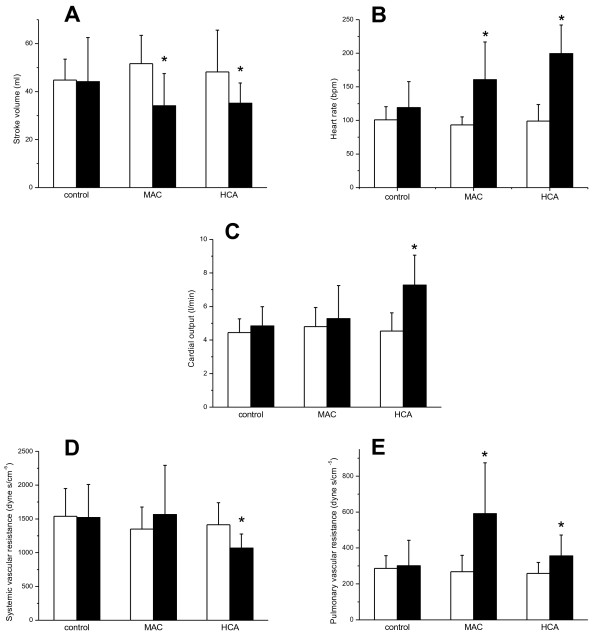
**Effects of acidosis on hemodynamics.** Empty columns, baseline. Solid columns, acidosis (or corresponding time point in control experiments). *P < 0.05. **(A)** Effects of MAC and HCA on the stroke volume. **(B)** Effects of MAC and HCA on the heart rate. **(C)** Effects of MAC and HCA on the cardiac output. **(D)** Effects of MAC and HCA on the systemic vascular resistance. **(E)** Effects of MAC and HCA on the pulmonary vascular resistance.

A small significant increase in the systemic MAP was induced by HCA, but not by MAC (Table 2). The mean pulmonary arterial pressure (MPAP) was increased by both MAC and HCA (Table 2). The systemic vascular resistance (SVR) was reduced by HCA only (Table 2; Figure 1). The pulmonary vascular resistance (PVR) was increased by both MAC and HCA (Table 2; Figure 1). Although a tendency was noted for a higher PVR in MAC, the intergroup comparison revealed no significant difference between PVR in either MAC or HCA. In both MAC and HCA, the global end-diastolic volume (GEDV) was not affected; however, the stroke volume was reduced (Table 2; Figure 1). This reduction was accompanied by an increase in heart rate (Table 2; Figure 1), again induced by both MAC and HCA. Consequently, the cardiac output was preserved in MAC, and even increased in HCA (Table 2; Figure 1). The single ventricular stroke work, either right or left, was not influenced by any kind of acidosis (Table 2). When the ventricular stroke work was calculated per minute, the right ventricular stroke work was increased by both MAC and HCA; however, the left ventricular stroke work was increased by HCA only (Table 2).

**Table 2:** General hemodynamics

	**Control**		** Metabolic acidosis**			**Respiratory acidosis**			
	**Baseline**_**st**_	**Baseline**_**end**_	**Δ**	**Baseline**	**Acidosis**	**Δ**	**Baseline**	**Acidosis**	**Δ**
HR	101 ± 20	119 ± 39		93 ± 12	161 ± 56	↑	99 ± 25	200 ± 42	↑
SV	44.8 ± 8.7	44.2 ± 18.4		51.6 ± 11.9	34.1 ± 13.4	↓	48.2 ± 17.4	35.2 ± 8.4	↓
GEDV	660.4 ± 116.2	691.1 ± 123.6		620.3 ± 136.6	571.8 ± 125.1		709.1 ± 152.9	693.7 ± 113.6	
CO	4.5 ± 0.8	4.8 ± 1.2		4.8 ± 1.1	5.3 ± 2.0		4.5 ± 1.1	7.3 ± 1.8	↑
MAP	94.5 ± 15.6	97.3 ± 16.9		87.4 ± 15.3	99.7 ± 18.4		88.7 ± 5.6	105.6 ± 9.4	↑
CVP	11.1 ± 2.1	10.6 ± 2.6		9.0 ± 2.6	9.3 ± 2.4		12.1 ± 3.8	12.1 ± 1.1	
MPAP	25.1 ± 5.2	26.1 ± 3.9		23.1 ± 3.1	42.9 ± 8.4	↑	24.4 ± 1.7	40.3 ± 6.7	↑
PCWP	9.5 ± 1.9	9.3 ± 2.4		7.9 ± 2.5	9.3 ± 2.7		10.9 ± 1.2	9.3 ± 2.4	
SVR	1,539 ± 409	1,520 ± 489		1,350 ± 327	1,564 ± 729		1,412 ± 327	1,068 ± 209	↓
PVR	287 ± 71	301 ± 142		268 ± 92	591 ± 283	↑	259 ± 60	356 ± 116	↑
ITBV	825.5 ± 145.3	863.9 ± 154.5		796 ± 173.3	715.9 ± 168.9	↓	886.4 ± 191.1	867.1 ± 142	
LVSW	59.5 ± 17.2	52.8 ± 20		61.8 ± 17.9	45.6 ± 18		58.4 ± 24.9	55.4 ± 12.4	
LVSW_tot_	6,056 ± 1,606	6,183 ± 1,783		5,784 ± 1,924	7,179 ± 2,835		5,788 ± 1,368	11,124 ± 2,968	↑
RVSW	15.5 ± 4.5	13.7 ± 4.5		16.3 ± 5.6	20.4 ± 10.8		16.7 ± 7.9	21.1 ± 7.3	
RVSW_tot_	1,606 ± 541	1,618 ± 419		1,492 ± 420	3,088 ± 1,414	↑	1,650 ± 539	4,166 ± 1,313	↑
HVSW	7,662 ± 2,041	7,801 ± 2,146		7,276 ± 2,201	10,267 ± 4,086		7,438 ± 1,874	15,290 ± 4,135	↑
Hearte	417.6 ± 111.2	425.2 ± 117		396.6 ± 120	559.6 ± 222.7		405.4 ± 102.1	833.4 ± 225.4	↑

↑, significantly increased, *P* < 0.05; ↓, significantly decreased, *P* < 0.05; HR, heart rate (bpm); SV, stroke volume (ml); GEDV, global end-diastolic volume (ml); CO, cardiac output (L/min); MAP, mean arterial pressure (mm Hg); CVP, central venous pressure (mm Hg); MPAP, mean pulmonary artery pressure (mm Hg); PCWP, pulmonary capillary wedge pressure (mm Hg); SVR, systemic vascular resistance (dyne s/cm^-5^); PVR, pulmonary vascular resistance (dyne s/cm^-5^); ITBV, intrathoracic blood volume (ml); LVSW, left ventricular stroke work (cJ), calculated as 0.0136 × MAP × SV; LVSW_tot_, left ventricular stroke work per minute (cJ/min); RVSW, right ventricular stroke work (cJ), calculated as 0.0136 × MPAP × SV; RVSW_tot_, right ventricular stroke work per minute (cJ/min); HVSW, (complete) heart ventricular stroke work per minute (cJ/min); heartE, heart energy (kcal).

Analysis of the regional hemodynamics revealed the following changes (Table 3): neither the total renal blood flow nor the renal fractional flow (renal blood flow as a fraction of cardiac output) changed significantly in either type of acidosis. Whereas the hepatic arterial blood flow was not affected by any type of acidosis, the portal venous blood flow and, in parallel, the total liver blood flow increased significantly in the pigs challenged with HCA only. The liver blood flow as a fraction of the cardiac output was reduced in both interventional groups. HCA significantly increased the blood flow through the carotid artery.

**Table 3:** Regional hemodynamics

	**Control**		** Metabolic acidosis**			**Respiratory acidosis**			
	**Baseline**_**st**_	**Baseline**_**end**_	**Δ**	**Baseline**	**Acidosis**	**Δ**	**Baseline**	**Acidosis**	**Δ**
Q_ren_/kg	5.5 ± 2.3	5.9 ± 2.4		5.1 ± 1.5	4.5 ± 1.1		4.7 ± 2.7	5.7 ± 3.5	
Q_ren_/Q_tot_	4.5 ± 3.2	4.5 ± 3.2		3.6 ± 1	3.4 ± 2.1		3.4 ± 1.7	2.6 ± 1.3	
Q_hep_/kg	2.9 ± 1.5	3 ± 1.6		2.8 ± 1.8	3.5 ± 1.8		4.8 ± 3	4.3 ± 2.1	
Q_port_/kg	27.4 ± 5.8	28.7 ± 6.1		30.0 ± 4.6	27.0 ± 5.5		25.5 ± 5.7	30.3 ± 6.7	↑
Q_liver_/kg	30.2 ± 6.3	31.7 ± 6.5		32.9 ± 5.4	30.4 ± 6.4		29.8 ± 7.5	34.0 ± 7.2	↑
Q_liver_/Q_tot_	23.6 ± 9.3	23.2 ± 9.4		23.4 ± 5	20.7 ± 5.8	↓	21.8 ± 3.5	16.1 ± 5.1	↓
Q_carot_/kg	4.6 ± 1.5	4.3 ± 1.2		5.3 ± 3.5	3.8 ± 1.2		5.0 ± 2.4	8.4 ± 3.7	↑
Q_carot_/Q_tot_	3.5 ± 1.1	3.1 ± 1		4.0 ± 3.1	2.8 ± 1.9		3.5 ± 1.3	3.6 ± 0.9	

↑, significantly increased, *P* < 0.05; ↓, significantly decreased, *P* < 0.05; Q_ren_/kg, renal blood flow per kg of body weight per min (ml/kg/min); Q_ren_/Q_tot_, fractional (fraction of cardiac output) renal blood flow (%); Q_hep_/kg, hepatic arterial blood flow per kg of body weight per min (ml/kg/min); Q_port_/kg, portal venous blood flow per kg of body weight per min (ml/kg/min); Q_liver_/kg, total liver blood flow per kg of body weight per min (ml/kg/min); Q_liver_/Q_tot_, fractional (fraction of cardiac output) liver blood flow (%); Q_carot_/kg, carotid arterial blood flow per kg of body weight per min (ml/kg/min); Q_carot_/Q_tot_, fractional (fraction of cardiac output) carotid arterial blood flow (%).

**Figure 2:**
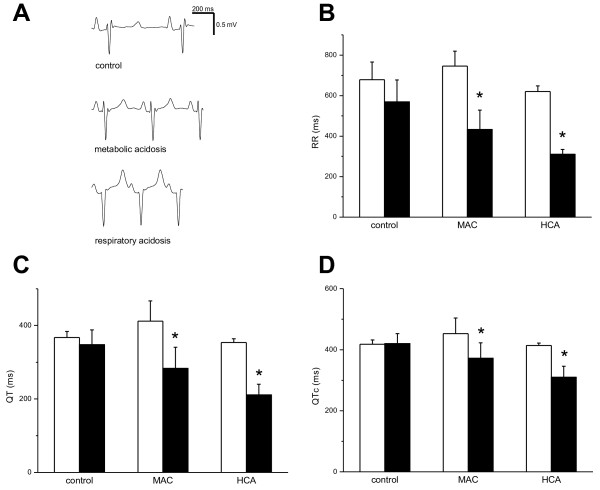
**Effects of acidosis on electrocardiogram.** Empty columns, baseline. Solid columns, acidosis (or corresponding time point in control experiments). *P < 0.05. **(A)** Representative electrocardiogram in control animal (upper trace), animal with MAC (middle trace), and animal with HCA (lower trace). **(B)** Effects of MAC and HCA on the RR interval. **(C)** Effects of MAC and HCA on the QT interval. **(D)** Effects of MAC and HCA on the QT_c_ interval (corrected by the Fridericia formula).

The electrocardiogram revealed a significant shortening of the QT interval in both MAC and HCA (Figure 2A, C). This effect was, in part, because of an increase in the heart rate (Figure 2B). A significant shortening remained present after a correction for the heart rate by the Fridericia formula (QT_c_ interval, Figure 2D). No arrhythmia was observed during any of the experiments. The analysis of the heart-rate variability revealed heterogeneous baseline characteristics within the experimental groups, with no significant difference between the groups, either before or after the intervention. More important, neither acidosis state was able significantly to affect the low-frequency (LF) or high-frequency (HF) components of the heart-rate oscillations that are generally accepted to represent the sympathovagal balance (for example, in HCA, the normalized LF parameters were 29.3 ± 14.3 before and 45.8 ± 19.3 after the induction of acidosis, and the normalized HF parameters were 70.7 ± 14.3 before and 54.2 ± 19.3 after the induction of acidosis).

**Figure 3:**
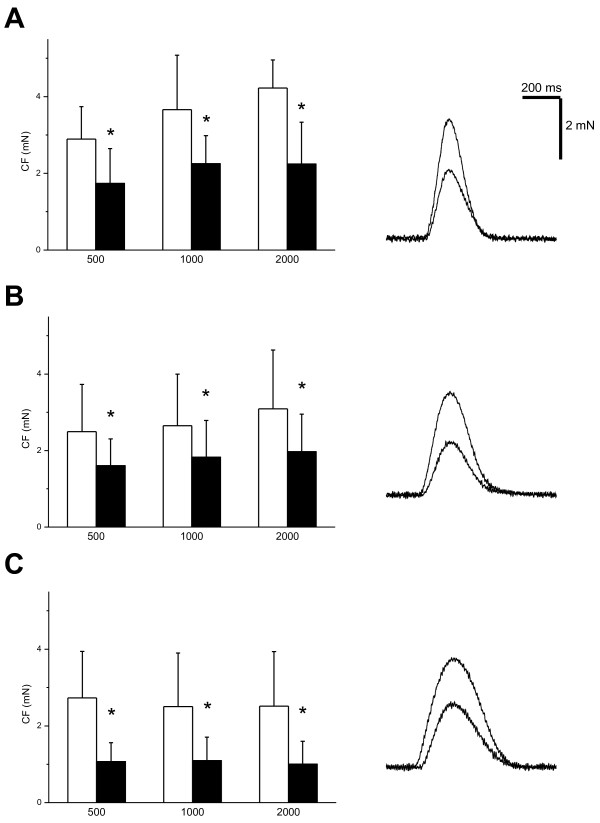
**Effects of acidosis on contraction of ventricular trabeculae.** Empty columns, control Tyrode solution. Solid columns, acidic solution (Tyrode solution with a pH of 7.1). *P < 0.05. **(A)** Effects of acidic solution on contraction force in trabeculae from control pigs (cycle length of 500, 1,000, and 2,000 ms). Left panel, pooled data. Right panel, representative contraction traces at stimulation frequency of 1 Hz in control and acidic solutions. **(B)** Effects of acidic solution on contraction force in trabeculae from pigs with MAC (cycle length of 500, 1,000, and 2,000 ms). Left panel, pooled data. Right panel, representative contraction traces at stimulation frequency of 1 Hz in control and acidic solutions. **(C)** Effects of acidic solution on contraction force in trabeculae from pigs with HCA (cycle length of 500, 1,000, and 2,000 ms). Left panel, pooled data. Right panel, representative contraction traces at stimulation frequency of 1 Hz in control and acidic solutions.

*In vitro*, in the right ventricular trabeculae, the baseline contraction force was not different between the control and acidic groups (Figure 3), regardless of the stimulation frequency (0.5, 1, or 2 Hz). The application of the acidic solution reduced the contraction force in all three groups (Figure 3), consistent with the reduction of stroke volume observed *in vivo*. The kinetics of the contraction-relaxation cycle were not influenced by acidosis. The time to the peak of contraction (TTP), as well as the time to 90% relaxation (R_90_), were similar in all three groups and were not influenced by the application of the acidic solution (for example, at a stimulation frequency of 1 Hz) in the trabeculae from the control pigs at a TTP of 127 ± 6 milliseconds and R_90_ of 147 ± 23 milliseconds in the control solution, and TTP of 126 ± 9 milliseconds and R_90_ of 141 ± 14 milliseconds in the acidic solution; in the trabeculae from the pigs with metabolic acidosis at a TTP of 135 ± 23 milliseconds and R_90_ of 164 ± 37 milliseconds in the control solution, and TTP of 123 ± 20 milliseconds and R_90_ of 161 ± 35 milliseconds in the acidic solution; or in the trabeculae from the pigs with respiratory acidosis at a TTP of 152 ± 14 milliseconds and R_90_ of 184 ± 54 milliseconds in the control solution, TTP of 140 ± 29 milliseconds and R_90_ of 170 ± 53 milliseconds in the acidic solution.

**Figure 4:**
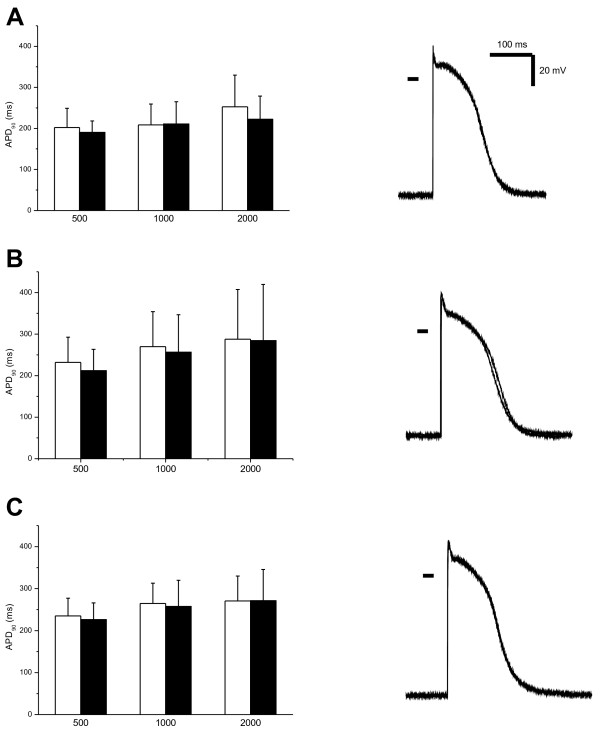
**Effects of acidosis on ventricular action potential.** Empty columns, control Tyrode solution. Solid columns, acidic solution (Tyrode solution with a pH of 7.1). **(A)** Effects of acidic solution on action potential in trabeculae from control pigs (cycle length of 500, 1,000, and 2,000 ms). Left panel, pooled data, APD_90_. Right panel, representative action potentials at stimulation frequency of 1 Hz in control and acidic solutions. **(B)** Effects of acidic solution on action potential in trabeculae from pigs with MAC (cycle length of 500, 1,000, and 2,000 ms). Left panel, pooled data, APD_90_. Right panel, representative action potentials at stimulation frequency of 1 Hz in control and acidic solutions. **(C)** Effects of acidic solution on action potential in trabeculae from pigs with HCA (cycle length of 500, 1,000, and 2,000 ms). Left panel, pooled data, APD_90_. Right panel, representative action potentials at stimulation frequency of 1 Hz in control and acidic solutions.

The membrane potential recordings revealed no effects of acidosis on the membrane electrogenesis; neither the resting membrane potential levels nor the action potentials were influenced by acidosis. The action potentials were of a similar shape, amplitude, and duration (APD_50_, APD_90_) in all three groups, and the application of the acidic solution did not exert any effects (Figure 4). The lack of the effect of acidosis on the action potential was observed at all stimulation frequencies tested (0.5, 1, and 2 Hz). Consistent with the *in vivo* experiments, no signs of cellular proarrhythmic events (action potential prolongation, triangulation, increased variability of repolarization, early and/or delayed afterdepolarizations) were observed *in vitro*.

## Discussion

This study documents that systemic acidosis, both HCA and MAC, exerts significant effects on the cardiovascular system. In the pulmonary circulation, the effects of both types of acidosis were similar: increased pulmonary vascular resistance and mean pulmonary artery pressure. The systemic circulation was affected differentially: whereas MAC did not influence the systemic hemodynamic parameters, HCA increased the mean arterial pressure, despite a reduction in the systemic vascular resistance. Both types of acidosis were associated with a decreased stroke volume and an increased heart rate.

Similar effects of acute respiratory acidosis (increase in cardiac output, decline in peripheral resistance) were reported previously in dogs [21]. In the same study, however, acute metabolic acidosis showed similar effects on both cardiac output and peripheral resistance. This discrepancy with our results may be attributed either to the mode of the metabolic acidosis induction (lactic acid versus hydrochloric acid), or to the species-specific differences (dog versus pig). In humans, an administration of CO_2_ produced an increase in cardiac output, stroke volume and heart rate, together with a reduction of the peripheral resistance [22]. This is in good agreement with our results, with the exception of the increase in stroke volume. This difference is probably related to the level of hypercapnia that was much lower in the study with human volunteers (the mean increase in P_a_CO_2_ was 11.5 torr).

These data indicate that in the pulmonary circulation, the main stimulus is the hydrogen ion concentration, because the effects of both types of acidosis were similar. This interpretation is supported by earlier studies in isolated cat lungs [11], isolated rat lungs [12], as well as in healthy humans [13]. Nevertheless, significant species differences may exist, because in the isolated rabbit lung, no effect of HCA on the normoxic pulmonary vascular tone was found [14]. The contributions of CO_2_ in our experiments cannot be excluded, because a significant increase in the partial pressure of CO_2_ in the venous blood was found for both types of acidosis; however, in MAC, it was much less pronounced.

Conversely, the lack of the effects of MAC in the systemic circulation strongly suggests that CO_2_ is a dominant stimulus for the systemic vasculature. The heart itself seems to be affected, preferentially, by hydrogen ions, as both types of acidosis were associated with similar changes in the cardiac function. To distinguish the direct cardiac effects from those induced secondarily because of primary vascular effects, the *in vitro* experiments with isolated cardiac tissues (right ventricular trabeculae) were performed, and they confirmed the *in vivo* findings. In agreement with the *in vivo* reduction of stroke volume, the contraction force of the trabeculae was decreased in the acidic solution.

A similar reduction of the myocardial contractility by hypercapnic acidosis was observed in the isolated perfused rat heart [23]. In contrast to that in our study, however, this cardiac effect was attributed to hypercapnia rather than to acidosis. Conversely, in a number of studies, the negative inotropic effects of acidosis *per se* were demonstrated (for example, see [7-9,24]). The diversity of earlier reports with regard to the effects of hypercapnia versus acidosis is probably related to differences in the experimental design (for example, differences in the time courses of the effects) and the experimental animal species.

The reduction in the contraction force was present at all stimulation frequencies tested (0.5, 1, and 2 Hz), thus arguing against the increase in the heart rate observed *in vivo* as a possible underlying factor. The heart rates reached *in vivo*, however, were substantially higher (up to 200 bpm), and the short filling times in such a tachycardia may significantly contribute to the reduction in the stroke volume. The lack of change in the global end-diastolic volume clearly indicates that the cardiac filling was not affected.

Shortening of the QT_c_ interval suggests that a shortening of the cardiac APD, associated with a reduction of the calcium influx into the cell through ICaL channels, may contribute to the impaired contractile function. To test this hypothesis, cardiac action potentials were measured *in vitro*, and no effects of acidosis were found. Therefore, the membrane electrogenesis and the trans-sarcolemmal calcium influx are not likely to contribute to the reduction in the contraction force. The discrepancy between the effects of acidosis on the cardiac repolarization *in vivo* (that is, shortening of the QT_c_ interval) and *in vitro* (that is, no effect on the action potential) may be related to a complex humoral and nervous regulation present *in vivo*, but not *in vitro*, and/or to inherent limitations in the QT-interval correction. Any correction formula (including the Fridericia formula used in this study) is likely to introduce an error [25].

Both HCA and MAC were associated with an increase in the heart rate. Such an increase may be due to autonomic cardiac regulation, or due to the direct effect of acidosis on sinoatrial-node cells. In isolated rabbit sinoatrial nodes, a negative chronotropic effect of the acidosis was described, and it was attributed to the protonation of ionic channels [26]. Similar results were obtained in canine sinoatrial node tissues [27]. In our experimental conditions, the absence of any effect of acidosis on the membrane (action) potential in ventricular tissues indicates that a major effect of acidosis on electrogenesis in the sinoatrial node is unlikely. These results suggest that the observed increase in the heart rate is related to the autonomic cardiac regulation.

A sympathetic neural activation, by acute HCA with increased plasma levels of both norepinephrine and epinephrine, *in vivo* in swine, was reported [28]. However, a stimulated norepinephrine release directly in the heart was shown to be reduced by acidosis [29,30], but only at pH values of 6.4 and lower. At a pH of 7.0, no acidosis-induced reduction in norepinephrine release was observed, but a pronounced suppression of the presynaptic muscarinic inhibition of norepinephrine release was identified [30]. Therefore, the modulation of the presynaptic muscarinic inhibition of norepinephrine release, and/or the increased plasma levels of catecholamines, may be the major mechanisms of the acidosis-induced increase in the heart rate. Our analysis of the heart-rate variability, revealing no shift in the sympathovagal balance (no change in LF and HF components or their ratios), argues against a general elevation of the plasma levels of catecholamines, and suggests a more-complex feedback mechanism (such as a presynaptic inhibition) being involved. This view is supported by a recent study [31], in which a presynaptic autoregulatory feedback mechanism was suggested to explain the paradoxic decrease in LF oscillations (that normally reflect the magnitude of the sympathetic activation) in conditions of elevated levels of circulating norepinephrine.

Besides the peripheral effects of acidosis, the role of the central chemoreceptors in the medulla should be considered. The high sensitivity of the central chemoreceptors to blood pCO_2_ could contribute to the increase in the cardiac output induced by HCA (but not MAC).

A reduction in cardiac contractility was confirmed *in vitro* in isolated preparations. A major contribution of the autonomic regulation to the negative inotropic effect is, therefore, unlikely, and a direct effect of acidosis on the processes of excitation-contraction coupling must be further scrutinized. The membrane electrogenesis was insensitive to the acidosis: at the pH of 7.1, no changes in the cardiac action potential were found. At this pH, the ionic currents (including ICaL) are not influenced, and their modification (for example, a reduction of ICaL) does not contribute to the reduced contractility. Most likely, intracellular acidosis develops and affects a number of intracellular mechanisms involved in calcium handling and contraction; for example, a deactivation of contractile proteins including impaired calcium binding to troponin C, impaired interaction of the troponin-tropomyosin complex, and impaired actin-myosin interactions [8,32,33]. It should be emphasized that the results of these studies were obtained at a more-pronounced acidosis level (pH about 6.5), corresponding to cardiac ischemia, and it is unclear whether these mechanisms (and to what extent) contribute at the clinically relevant systemic acidosis addressed in this study (plasma pH 7.1).

The right ventricular stroke work per minute was increased by both types of acidosis; however, the left ventricular stroke work per minute was increased by HCA only. Therefore, acidosis places an increased work demand on the heart: MAC (perhaps) on the right ventricle only, and HCA on both ventricles. This finding may substantially weaken the emerging paradigm of therapeutic HCA, especially in conditions of compromised cardiac function. Although a number of possibly beneficial effects of HCA, including antiinflammatory effects [2], were reported recently, in conditions of (right ventricular) heart failure and/or pulmonary hypertension, considerable caution is warranted. It should be noted that the negative inotropic effect was accompanied by an increased heart rate to maintain (MAC), or even increase (HCA), the cardiac output. Consequently, the development of acidosis (although in our conditions of HCA, with rather extreme values of pCO_2_) may be especially dangerous in clinical conditions that are associated with an elevated heart rate (for example, sepsis), in which an additional increase in heart rate is limited, and therefore, the cardiac output cannot be maintained.

In sharp contrast to the uniform effects of both types of acidosis on pulmonary circulation, we observed quite a heterogeneous response in the hepato-splanchnic region. We are not aware of any other study that simultaneously compares the effects of both MAC and HCA on multiple vascular beds. First, the lack of a measurable effect of HCA on the global renal blood flow is in contrast to several previous studies reporting both increased [34] and reduced renal perfusion, in response to acute hypercapnia [35]. Second, our results suggest that the liver circulation might behave differently in subjects exposed to HCA (increased) as opposed to MAC (unchanged). Interestingly, HCA increased the portal venous flow without affecting the hepatic arterial blood flow. Taken together, these findings suggest that the vasodilatory effect of carbon dioxide on the hepato-splanchnic circulation is largely independent of the changes in pH, and indicate the different effects of HCA on the arterial versus portal venous blood supply in the liver. Nonetheless, the exact mechanism and clinical importance of these physiological responses cannot be answered from the present data.

### Limitations of the study

The intracellular mechanisms underlying the negative inotropic and chronotropic cardiac effects, as well as the vascular effects, were not addressed in this study. Instead, the study was oriented toward integrative physiology, with an emphasis on clinically relevant *in vivo* phenomena, which were verified and further elucidated in experiments with isolated tissues.

We assessed the effects of acidosis at only a single pH level. Therefore, the dose-effect relation could not be established.

The level of hypercapnia needed to achieve a pH of 7.1 was relatively high in our study, thereby mimicking severe acute exacerbation of chronic obstructive pulmonary disease, rather than permissive hypercapnia in ARDS. It is of note that the rather high values of pCO_2_ limit the clinical relevance of this study, and that in conditions of milder (permissive) hypercapnia, the beneficial effects may prevail. Because of the relatively short-term duration of our experiment, our observations may not apply to prolonged acidosis.

The distal organ-perfusion experiments were performed by using probes placed around the major arteries, and consequently, only information about the total organ perfusion was obtained. Possible changes in small arteries-arterioles may not have been detected.

In the *in vitro* experiments with cardiac trabeculae, only the effects of (metabolic) acidosis *per se* were tested. The perfusion solutions with a pH of 7.4 or 7.1, with no CO_2_ present, were used for preparations from all three experimental groups.

Although no signs of cardiac arrhythmic and/or proarrhythmic events were observed either *in vivo* (ECG analysis) or *in vitro* (cellular membrane potential recordings), a change in the susceptibility to cardiac arrhythmias cannot be excluded. A detailed examination of the cardiac arrhythmic susceptibility with pharmacologic and/or electrophysiological challenges is, however, beyond the scope of this study.

## Conclusions

Clinically relevant acidosis, both HCA and MAC, affects the cardiovascular system in a very complex and heterogeneous way. MAC preferentially affects the pulmonary circulation, whereas HCA affects the pulmonary, systemic, and regional circulations. The cardiac contractile function was reduced, but the cardiac output was maintained (MAC), or even increased (HCA). The increased ventricular stroke work per minute revealed an increased work demand placed by acidosis on the heart. Based on the lack of the effect of acidosis on the action potential, the processes of membrane electrogenesis are, probably, not involved in the cardiac effects of clinically relevant acidosis. The cardiovascular effects of acidosis may limit the possible therapeutic use of HCA, especially in the setting of compromised cardiac function.

## Key messages

• HCA affects pulmonary, systemic, and regional circulations, in contrast to MAC, which preferentially affects the pulmonary circulation.

• Acidosis diminishes cardiac contractility and increases the ventricular stroke work per minute.

• Acidosis places an increased work demand on the heart, which may limit the therapeutic potential of HCA.

• The organ-specific response to HCA appears not to be uniform.

## Abbreviations

ARDS: Acute respiratory distress syndrome; BEa: Base excess in arterial blood; BEv: Base excess in venous blood; CO: Cardiac output; CVP: Central venous pressure; GEDV: Global end-diastolic volume; Hb: Hemoglobin concentration; HCA: Hypercapnic acidosis; $\mathrm{HC}O_{3}{}_{a}^{-}$: $\mathrm{HC}O_{}{}_{3}^{–}$ concentration in arterial blood; $\mathrm{HC}O_{3}{}_{v}^{-}$: $\mathrm{HC}O_{}{}_{3}^{–}$ concentration in venous blood; heartE: Heart energy; HR: Heart rate; HVSW: (Complete) Heart ventricular stroke work per minute; ITBV: Intrathoracic blood volume; LVSW: Left ventricular stroke work; LVSWtot: Left ventricular stroke work per minute; MAC: Metabolic acidosis; MAP: Mean arterial pressure; MPAP: Mean pulmonary artery pressure; PaCO2: Arterial partial pressure of CO_2_; PaO2: Arterial partial pressure of O_2_; PCWP: Pulmonary capillary wedge pressure; pHa: Arterial pH; pHv: Venous pH; PvCO2: Venous partial pressure of CO_2_; PvO2: Venous partial pressure of O_2_; PVR: Pulmonary vascular resistance; Qcarot/kg: Carotid arterial blood flow per kilogram of body weight per minute; Qcarot/Qtot: Fractional (fraction of cardiac output) carotid arterial blood flow; Qhep/kg: Hepatic arterial blood flow per kilogram of body weight per minute; Qliver/kg: Total liver blood flow per kilogram of body weight per minute; Qliver/Qtot: Fractional (fraction of cardiac output) liver blood flow; Qport/kg: Portal venous blood flow per kilogram of body weight per minute; Qren/kg: Renal blood flow per kilogram of body weight per minute; Qren/Qtot: Fractional (fraction of cardiac output) renal blood flow; RVSW: Right ventricular stroke work; RVSWtot: Right ventricular stroke work per minute; Sata: Hemoglobin saturation in arterial blood; Satv: Hemoglobin saturation in venous blood; SV: Stroke volume; SVR: Systemic vascular resistance; TTP: Time to the peak of contraction.

## Competing interests

The authors declare that they have no competing interests.

## Authors’ contributions

MS participated in the electrophysiological studies, in the coordination of the study, performed the statistical analyses, and drafted the manuscript. LL participated in the *in vivo* hemodynamic studies and performed the statistical analyses. JC participated in the *in vivo* hemodynamic studies, in the coordination of the study, and performed the statistical analyses. JB participated in the *in vivo* hemodynamic studies and performed the statistical analyses. DJ developed the Matlab routines, analyzed the heart-rate variability, and performed the statistical analyses. JH participated in the electrophysiological studies. PS participated in the electrophysiological studies. JS participated in the electrophysiological studies. MM conceived of and designed the study, participated in the coordination of the study, and helped to draft the manuscript. All authors read and approved the final manuscript.
